# Design of a Novel Polyvinyl Imidazole-Based Adsorbent for Efficient Textile Dye Removal

**DOI:** 10.3390/nano15221708

**Published:** 2025-11-12

**Authors:** Seyda Tugba Gunday, Arkan Almushikes, Fatmah Al Bibiy, Noor Alzayer, Lama Almedaires, Aljawharah Alagl, Ismail Anil, Omer Aga

**Affiliations:** 1 Bioenergy Research Unit, Department of Biophysics, Institute for Research and Medical Consultations (IRMC), Imam Abdulrahman Bin Faisal University, P.O. Box 1982, Dammam 34212, Saudi Arabia; 2 Environmental Engineering Department, College of Engineering A13, Imam Abdulrahman Bin Faisal University, P.O. Box 1982, Dammam 34212, Saudi Arabia; mushakiesarkan@gmail.com (A.A.); albibifatima@gmail.com (F.A.B.); noor.alzayer15@gmail.com (N.A.); lamaalmedaires@hotmail.com (L.A.); aljawharahasa@gmail.com (A.A.); oaga@iau.edu.sa (O.A.)

**Keywords:** polyvinyl imidazole, procion red, textile dye, dye removal, dye adsorption, wastewater treatment

## Abstract

Textile dye effluents containing toxic organic compounds pose serious environmental challenges. In this study, novel Poly(1-vinyl imidazole)-Bis[2-(methacryloyloxy)ethyl] phosphate (PVIB) polymers were synthesized with crosslinker molar fractions ranging from 5% to 80% and were subsequently investigated as advanced adsorbents for textile dye removal. Procion Red (PR), a widely used reactive dye, was selected as the model pollutant. The materials were characterized using FTIR, TGA, DTG, SEM-EDX, WD-XRF, TEM, and BET analyses. Adsorption mechanisms were examined through kinetic, isotherm, and thermodynamic models. Among the synthesized formulations, PVIB20% achieved the best dye removal, reaching an experimental adsorption capacity of 330 mg g^−1^ within 60 min under acidic to neutral conditions. The kinetic modeling studies identified the pseudo-first-order model as the best fit, indicating a surface-controlled process involving both physical and chemical interactions. Isotherm studies showed that the Langmuir and Redlich–Peterson models provided the best fit, yielding a maximum monolayer adsorption capacity of 765 mg g^−1^. Thermodynamic analysis revealed that the adsorption was spontaneous, endothermic, and entropy-driven. Overall, PVIB20% demonstrated superior adsorption capacity, rapid kinetics, and strong dye–polymer interactions compared with many conventional and modified adsorbents, which highlights its potential as an efficient and durable material for anionic dye removal from wastewater.

## 1. Introduction

Dye-containing wastewaters represent a significant environmental challenge due to their complex composition of synthetic dyes, toxic chemicals, and heavy metals. Textile industries are the primary contributors, discharging millions of liters of dye-laden effluents annually into natural water bodies [1]. Synthetic dyes, particularly azo and reactive dyes, are characterized by their high stability, resistance to degradation, and intense coloration, which impede light penetration in aquatic ecosystems [2]. This disruption inhibits photosynthesis and leads to oxygen depletion and hypoxic conditions that threaten aquatic biodiversity. Furthermore, the persistence of dyes facilitates bioaccumulation and biomagnification along the food chain, ultimately posing risks to human health through carcinogenicity, mutagenicity, and organ toxicity [3,4]. Dyes cause visible coloration even at low concentrations (<1 mg L^−1^) and reduce water quality and aesthetic value. The persistent nature of these pollutants limits the effectiveness of conventional biological treatment methods. Given the scale and persistence of this pollution, effective remediation strategies are critical to mitigate ecological risks and comply with increasingly stringent environmental regulations [5,6].

Various methods for dye wastewater treatment have been employed, including physical, chemical, and biological processes [7,8]. Physical methods such as coagulation, flocculation, membrane filtration, and adsorption primarily rely on phase separation but often face limitations regarding cost and sludge generation [9]. Chemical approaches, including advanced oxidation processes such as ozonation, photocatalysis, and Fenton reactions, achieve high decolorization efficiencies by mineralizing dye molecules into simpler compounds. However, they can be energy-intensive, produce toxic by-products, and require precise operational control [10,11,12]. Biological methods, using bacteria, fungi, or algae, are cost-effective and eco-friendly but generally ineffective against synthetic dyes due to their complex aromatic structures and resistance to biodegradation [13,14]. Among these, adsorption has emerged as the most promising technique due to its operational simplicity, cost-effectiveness, and high efficiency even at low concentrations. A wide variety of adsorbents have been investigated, ranging from activated carbon to low-cost materials such as agricultural residues, biochar, clays, and industrial by-products, which are also applicable to plant and cellulosic fibers [15,16,17,18].

Procion Red MX-5B, a widely used reactive dye in textile industries, has gained attention due to its toxicity, high solubility, and resistance to biodegradation. A wide range of adsorbents has been investigated for Procion Red MX-5B (PR) dye removal, focusing on efficiency, adsorption capacity, and sustainability. Agricultural waste such as peach pits and corncobs has been converted into activated carbons, achieving PR adsorption capacities up to 297.2 mg g^−1^ and effective removal within extended contact times [19,20]. Similarly, modified avocado shells demonstrated capacities of 167–212 mg g^−1^ with >90% PR dye removal efficiency under neutral pH [21]. Alumina–activated carbon composites showed reusability but reduced performance after repeated cycles [22]. Clay-based adsorbents, including organosilane-modified montmorillonite and bentonite, exhibited strong dye affinity, with maximum PR adsorption capacities of 318.6 mg g^−1^ and rapid uptake under optimized conditions [23,24]. Synthetic talc and kaolin also proved effective, yet with lower adsorption capacity (12.9 mg g^−1^) and longer equilibrium times [25]. Magnetite–humic acid layered double hydroxide achieved 192 mg g^−1^ adsorption capacity with excellent recyclability [26], while activated carbon–Fe_3_O_4_ composites reached 278 mg g^−1^ with 94% removal in just 50 min [27]. Adsorption of PR dye generally follows pseudo-second-order kinetics, indicating chemisorption, though some composites fit pseudo-first-order models. Equilibrium is best described by Langmuir or Sips models, suggesting monolayer coverage on homogeneous sites. Thermodynamic analyses consistently reveal spontaneous adsorption, with both exothermic and endothermic behaviors depending on adsorbent type. These studies highlight bio-based and composite adsorbents as efficient, low-cost, and sustainable alternatives for dye-bearing wastewater treatment.

Vinylimidazole (VI)-based polymers are promising materials for environmental remediation due to their imidazole rings, which can be protonated under acidic conditions to provide active sites for electrostatic attraction, hydrogen bonding, and coordination with pollutants. The vinyl group facilitates copolymerization, enabling tailored structures with improved stability, hydrophilicity, and functional diversity. Poly(1-vinylimidazole), poly(2-vinylimidazole), and related polyvinyl imidazole (PVI) derivatives exhibit high thermal and chemical stability, making them attractive for practical applications [28,29]. Vinyl imidazole-based adsorbents have attracted growing attention for dye removal due to their tunable functional groups, high charge density, and strong affinity for anionic and cationic dyes. Poly(N-vinylimidazole)-grafted pullulan hydrogels demonstrated pH-responsive adsorption, with maximum capacities of 36.6 mg g^−1^ for Methyl Orange and 5.72 mg g^−1^ for Acid Green 25 at pH 3, following pseudo-second-order kinetics and Freundlich isotherm fitting [30]. Similarly, xanthan gum/poly(vinylimidazole)-montmorillonite nanocomposites achieved nearly complete removal of malachite green (909.1 mg g^−1^) within 90 min, best described by the Langmuir model [31]. Ionic liquid-crosslinked polyacrylamide and poly(HEMA) hydrogels bearing vinylimidazole showed nearly 100 mg g^−1^ capacities for eosin B, with adsorption explained by Langmuir/Freundlich isotherms and pseudo-second-order kinetics [32]. Magnetic chitosan–vinylimidazole hydrogels reached very high uptake of methylene blue (860 mg g^−1^), fitting Langmuir and pseudo-second-order models, while maintaining >92% efficiency over five cycles [33]. PVI gel composite membranes exhibited rapid dye separation with capacities of 79 mg g^−1^ for Sunset Yellow and superior flux compared to nanofiltration [34]. Quaternized p(MAPTAC-co-VI) hydrogels showed record adsorption capacities (1818 mg g^−1^ for Eriochrome Black T and 1449 mg g^−1^ for Methyl Orange), with adsorption being spontaneous and endothermic [35]. Finally, PVI-grafted polymeric beads exhibited selective dye uptake, achieving 111 mg g^−1^ for Calcon and showing Langmuir isotherm behavior [36]. VI-based adsorbents exhibit versatile adsorption behaviors, typically following Langmuir-type monolayer chemisorption and pseudo-second-order kinetics, with many systems demonstrating high recyclability, rapid kinetics, and strong electrostatic interactions as the dominant mechanism.

In this study, novel Poly(1-vinyl imidazole)-Bis[2-(methacryloyloxy)ethyl] phosphate (PVIB) polymers were synthesized, characterized, and applied for dye removal for the first time. A widely used reactive PR dye was selected as a model pollutant to evaluate the performance of PVIB prepared with varying Bis[2-(methacryloyloxy)ethyl] phosphate ratios (5–80%). The structural and morphological properties of PVIB were characterized by Fourier transform infrared (FT-IR) spectroscopy, thermogravimetric analysis (TGA), derivative thermogravimetric analysis (DTG), scanning electron microscopy (SEM) with energy-dispersive X-ray spectroscopy (EDX), transmission electron microscopy (TEM), wavelength dispersive X-ray fluorescence (WD-XRF), surface area (BET) and pore size/volume (BJH) analysis techniques. Afterwards, the adsorption mechanisms were examined through kinetic, isotherm, and thermodynamic models. The novelty of this work lies in combining imidazole functionalities with phosphate-containing monomers to produce robust and multifunctional adsorbents with tunable surface chemistry. The study aims to achieve efficient and rapid dye removal with low adsorbent dosage, demonstrating strong potential for wastewater treatment in the textile sector.

## 2. Materials and Methods

### 2.1. Materials

The following chemicals were obtained from Merck© (KGaA, Darmstadt, Germany) and used without additional purification: 1-vinyl imidazole 99% (VI), Bis[2-(methacryloyloxy)ethyl] phosphate (B), toluene, azobisisobutyronitrile (AIBN), Procion red MX-5B (PR), sulfuric acid (H_2_SO_4_, 95–97%), and sodium hydroxide (NaOH) pellets. PR dye solutions, essential for both batch adsorption experiments and spectrophotometer calibration, were freshly prepared prior to their usage by dissolving the required amount of PR in ultrapure water, which was obtained using a Milli-Q© water purification system (Bedford, MA, USA).

### 2.2. Preparation of Poly (1-vinyl imidazole)-Bis[2-(methacryloyloxy)ethyl] Phosphate (PVIB)

**Figure 1:**
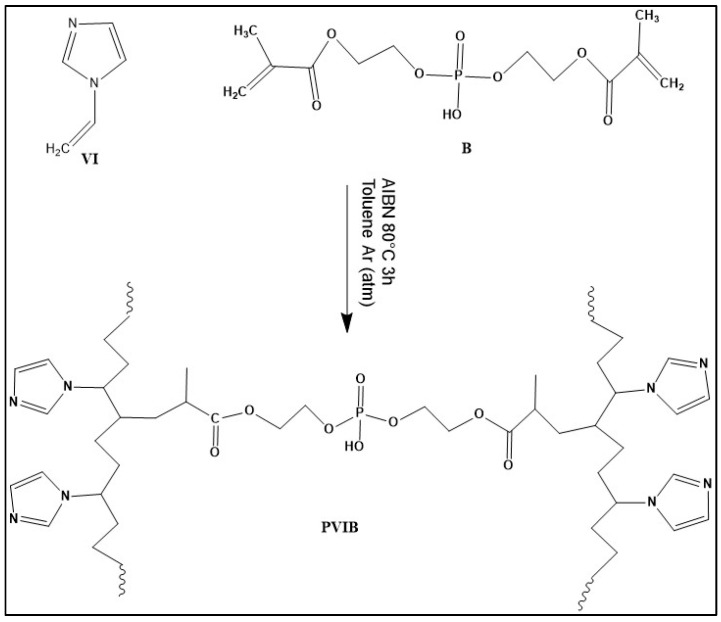
Schematic illustration for the synthesis of poly(vinylimidazole)-bis[2-(methacryloyloxy)ethyl] phosphate (PVIB).

Figure 1 illustrates the synthetic route for preparing the PVIB copolymer through a free radical polymerization process. The copolymer was synthesized by polymerizing 1-vinylimidazole (VI) in the presence of Bis[2-(methacryloyloxy)ethyl] phosphate (B) as a crosslinking agent and azobisisobutyronitrile (AIBN) as a thermal initiator. Upon heating, AIBN decomposes to generate free radicals, which initiate the polymerization of the vinyl groups in both VI and B, resulting in the formation of a crosslinked copolymer network that contains imidazole functionalities and phosphate groups.

The imidazole moieties provide active sites for interaction with dye molecules, while the phosphate units enhance the structural stability and hydrophilicity of the polymer network, making the resulting PVIB material suitable for adsorption applications and potential functional modifications. Several molar fractions of B (ranging from 5% to 80% relative to VI) were employed to investigate the effect of crosslinking density on the structural and adsorption properties. For example, PVIB (5%) was synthesized using 1.0 g of VI (0.01 mol), 0.16 g of B, 32 mg of AIBN (0.2 mmol), and 5 mL of toluene. The reaction mixture was placed in a Schlenk flask, degassed, and backfilled with argon, then polymerized at 80 °C for three hours. After polymerization, the product was thoroughly washed with ethanol to remove unreacted components. The insolubility of the crosslinked copolymer in water confirmed the formation of a stable three-dimensional network while maintaining hydrophilic functional groups on the surface. Finally, obtained copolymers were dried under vacuum for subsequent characterization and adsorption studies [16,37].

### 2.3. Characterization of PVIB Adsorbent

The FT-IR spectra of the PVIB, PVIB after PR dye adsorption (PVIBPR), and PR dye were recorded within the range of 400–4000 cm^−1^ at a resolution of 4 cm^−1^ at ambient temperature using an FT-IR spectrophotometer (Shimadzu IR Spirit, Kyoto, Japan). Thermogravimetric (TGA) and derivative thermogravimetric (DTG) analyses of PVIB, PVIBPR, and PR were performed using a simultaneous thermal analyzer (Mettler Toledo, Columbus, OH, USA). The measurements were conducted from 25 °C to 650 °C at a heating rate of 10 °C min^−1^ under a nitrogen atmosphere with a flow rate of 20 mL min^−1^. The DTG curves were obtained from the TGA data to determine the decomposition steps and corresponding weight-loss rates. The morphology of the PVIB and PVIBPR was examined using a scanning electron microscope (SEM, Inspect S50, FEI Company, Hillsboro, OR, USA), with samples being gold-coated prior to imaging. Detailed surface morphology, structural analysis, and observation of PVIB and PVIBPR were carried out using transmission electron microscopy (TEM, Morgagni 268, FEI Company, Brno, Czech Republic). The WD-XRF spectrometer (Rigaku ZSX Primus III+, Tokyo, Japan) was used to determine the elemental composition of the PVIB samples. The elements from F to U in the periodic table were scanned in the most sensitive mode of the instrument during WD-XRF analysis. The Brunauer–Emmett–Teller (BET) surface area, pore volume, and pore size of the materials were determined using a Quantachrome Nova 2200e instrument (Anton Paar GmbH, Graz, Austria), with samples degassed at 100 °C for 6 h in nitrogen. Multi-point BET measurements involved nitrogen adsorption/desorption at 77 K.

### 2.4. Adsorption Studies

Batch adsorption experiments were conducted in 50 mL amber glass Erlenmeyer flasks to minimize potential photodegradation of PR molecules. Each flask contained varying amounts of adsorbent (5–100 mg) and 50 mL of PR solution with initial concentrations ranging between 50 and 500 mg L^−1^. The mixtures were agitated on digital magnetic stirrers with heating (RTC5, IKA-Werke GmbH & Co. KG, Staufen im Breisgau, Germany) at 300 rpm and maintained at different temperatures (25, 35, 45, and 55 °C) for a contact time of 180 min. The initial solution pH was adjusted with 1 N NaOH or 1 N H_2_SO_4_. Aliquots were collected at scheduled intervals, centrifuged at 5000 rpm for 5 min, and the remaining PR concentration was quantified at 530 nm using a spectrophotometer (Hach-Lange DR 3900, Loveland, CO, USA). Calibration was performed for each experimental set using six PR standard solutions (5–100 mg L^−1^), yielding a linear calibration curve described as [PR] (mg L^−1^) = 57.7 × Absorbance (at 530 nm), with an R^2^ of 0.999. The amount of PR adsorbed per gram of adsorbent (q_t_, mg g^−1^) and the percentage removal efficiency (RE, %) at a given contact time were determined using Equations (1) and (2), respectively.

(1) $$q_{t}\left(mg\;g^{-1}\right)=\frac{\left(C_{0}-C_{t}\right)V}{M_{a}}$$

(2) $$RE\left(\%\right)=\frac{C_{0}-C_{t}}{C_{0}}\times 100$$

where C_0_ = initial PR concentration (mg L^−1^); C_t_ = PR concentration at time t (mg L^−1^); V = solution volume (L); M_a_ = adsorbent mass (g).

### 2.5. Adsorption Kinetics

The adsorption of dyes onto adsorbents typically proceeds through several sequential stages: (i) surface interaction with the adsorbent, (ii) diffusion across both the external and internal surfaces, and (iii) penetration and transport within the porous structure [38,39]. To evaluate the kinetics and mechanisms of PR adsorption onto PVIB polymers, four kinetic models were employed: the pseudo-first-order (PFO) [40], pseudo-second-order (PSO) [41], intraparticle diffusion (IPD) [42], and Elovich [43] models. The standard expressions of these models (Equations (3)–(6)), together with the corresponding plots used to determine their kinetic parameters, are presented in Table 1. In these equations, q_e_ (mg g^−1^) represents the adsorption capacity at equilibrium. The rate constants are defined as follows: k_1_ (min^−1^) for the PFO model, k_2_ (g mg^−1^ min^−1^) for the PSO model, and k_d_ (mg g^−1^ min^−0.5^) for the IPD model, with C (mg g^−1^) accounting for the boundary layer resistance. For the Elovich model, α (mg g^−1^ min^−1^) corresponds to the initial adsorption rate, while β (g mg^−1^) reflects the desorption-related constant.

**Table 1:** Kinetic models, their standard forms, and the corresponding plot types for calculating model parameters.

Kinetic Model	Standard Form	Plot	Equation No
Pseudo-first-order	$q_{t}=q_{e}(1-e^{k_{1}t})$	qt vs. t	(3)
Pseudo-second-order	$q_{t}=\frac{q_{e}^{2}k_{2}t}{1+q_{e}k_{2}t}$	qt vs. t	(4)
Intraparticle diffusion	$q_{t}=k_{d}t^{0.5}+C$	qt vs. t	(5)
Elovich	$q_{t}=\frac{1}{\beta }ln\left(1+\alpha \beta t\right)$	qt vs. t	(6)

### 2.6. Adsorption Isotherms

Investigating adsorption isotherms is essential for understanding the nature of interactions between dye molecules and the adsorbent surface and how dye species are distributed between liquid and solid phases at equilibrium [44,45]. Three commonly used isotherm models—Langmuir, Freundlich, and Redlich–Peterson—were applied to interpret the equilibrium data. The Langmuir model assumes that adsorption occurs as a uniform monolayer on a homogenous surface with identical active sites [46]. In contrast, the Freundlich model describes adsorption on heterogeneous surfaces, where multilayer adsorption can occur with varying site energies [47]. The standard expressions of the Langmuir and Freundlich equations are given in Equations (7) and (8), respectively:

(7) $$q_{e}=\;\frac{q_{m}\;K_{L}\;C_{e}}{1+\;K_{L}\;C_{e}}$$

(8) qe=KF Ce1n

In the isotherm equations, C_e_ (mg L^−1^) denotes the equilibrium concentration of PR remaining in solution, while q_m_ (mg g^−1^) refers to the theoretical maximum adsorption capacity of the adsorbent. The constant K_L_ (L mg^−1^) represents the Langmuir model parameter, whereas K_F_ (mg g^−1^) is the Freundlich isotherm constant, and 1/n is a dimensionless factor that reflects the adsorption intensity. In addition, the Langmuir model allows the calculation of a dimensionless parameter known as the separation factor or equilibrium parameter (R_L_), which indicates the favorability of the adsorption process, as expressed in Equation (9).

(9) $$R_{L}=\frac{1}{1+K_{L}C_{o}}$$

The value of R_L_ provides insight into the nature of the adsorption process: adsorption is considered irreversible when R_L_ = 0, favorable when 0 < R_L_ < 1, linear when R_L_ = 1, and unfavorable when R_L_ > 1. In addition to the Langmuir and the Freundlich models, the Redlich–Peterson isotherm is often applied. This empirical three-parameter model integrates characteristics of both Langmuir and Freundlich equations, making it suitable for describing adsorption behavior on either homogeneous or heterogeneous surfaces [48]. The standard form of the Redlich–Peterson isotherm is given in Equation (10).

(10) $$q_{e}=\frac{K_{rp}C_{e}}{1+\alpha_{rp}C_{e}^{\beta }}$$

In this model, K_rp_ (L g^−1^) and α_rp_ (L mg^−1^) represent the Redlich–Peterson constants, while β is a dimensionless exponent ranging between 0 and 1. When β = 1, the model reduces to the Langmuir isotherm, whereas for β = 0, it takes the form of the Freundlich isotherm.

### 2.7. Model Fit and Evaluation Metrics

The curve fitting of the experimental data to standard theoretical models was conducted using OriginPro software (ver. 2024). This program applies the least-squares algorithm, also known as chi-square minimization, to minimize the deviation between experimental data and theoretical predictions by iteratively selecting parameters that yield the smallest residuals. The Levenberg–Marquardt (L–M) algorithm, which combines the Gauss–Newton and steepest descent methods, was used to refine parameter values, ensuring stable convergence and accurate estimation of kinetic and isotherm parameters [49,50,51].

Goodness-of-fit and statistical validity were further evaluated using Marquardt’s percent standard deviation (MPSD), adjusted determination coefficient (adj-R^2^), reduced chi-square (χ^2^), Akaike Information Criterion (AIC), and Bayesian Information Criterion (BIC)—all calculated within OriginPro software. Statistical evaluation metrics such as MPSD, AIC, and BIC can be calculated using Equations (11)–(13), respectively. MPSD and adj-R^2^ indicate the closeness between experimental and modeled values, while χ^2^ measures the normalized deviation of the fit. AIC and BIC assess model adequacy by balancing accuracy with parameter complexity. The model exhibiting the lowest MPSD, reduced-χ^2^, AIC, and BIC values and the highest adj-R^2^ was considered the best representation of adsorption kinetics and equilibrium behavior.

(11) $$MPSD=100\sqrt{\frac{\sum {\left[\left(q_{e,exp}-q_{e,cal}\right)/q_{e,exp}\right]}^{2}}{n-1}}$$

(12) $$AIC=n\ln\left(\frac{RSS}{n}\right)+2k$$

(13) $$BIC=n\ln\left(\frac{RSS}{n}\right)+k\ln\left(n\right)$$

where q_e,exp_ (mg g^−1^) refers to the adsorption capacity determined experimentally for the PVIB polymers, whereas q_e,cal_ (mg g^−1^) denotes the adsorption capacity estimated by the isotherm model. The parameter n represents the total number of experimental observations considered in the analysis. RSS is the residual sum of squares, and k is the number of model parameters.

### 2.8. Adsorption Thermodynamics

The feasibility and underlying mechanism of adsorption can be elucidated through the evaluation of thermodynamic parameters, namely the standard Gibbs free energy change (ΔG^0^), standard enthalpy change (ΔH^0^), and standard entropy change (ΔS^0^). These parameters were determined using the Van’t Hoff approach, as expressed in Equations (14) and (15) [52].

(14) $$\ln\left(K_{D}\right)=-\frac{{∆H}^{0}}{R}\frac{1}{T}+\frac{{∆S}^{0}}{R}$$

(15) $${∆G}^{0}={∆H}^{0}-T{∆S}^{0}$$

The thermodynamic equilibrium constant (K_D_) was obtained from a plot of ln(q_e_/C_e_) versus q_e_, extrapolated to zero [53]. R denotes the universal gas constant (8.314 J mol^−1^ K^−1^), and T is the absolute temperature. Subsequently, values of ΔH^0^ and ΔS^0^ were derived from the slope and intercept of the linear plot of ln(K_D_) against 1/T.

## 3. Results and Discussion

### 3.1. Characterization of PVIB Adsorbents

#### 3.1.1. Fourier Transform Infrared (FT-IR) Spectra

**Figure 2:**
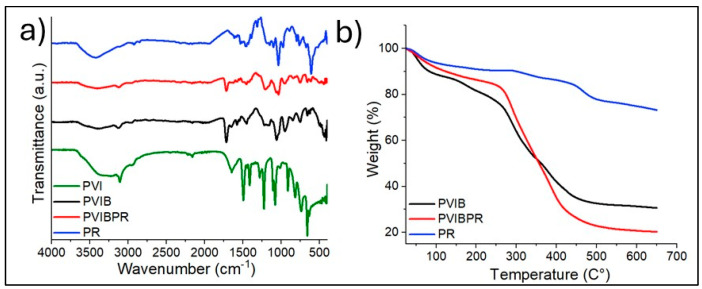
(**a**) FT-IR spectra of the PVIB, PVIBPR, PR. (**b**) TGA curves of PVIB, PVIBPR, PR.

The FTIR spectra of PVI and PVIB confirm the presence of characteristic functional groups and the successful formation of the composite Figure 2a. For PVI, absorption peaks at 3130–3050 cm^−1^ are associated with C–H stretching vibrations of the imidazole ring, while the band at 1630 cm^−1^ corresponds to C=N stretching, indicating the presence of imidazole functionalities [37]. In PVIB, additional peaks appear at approximately 1720 cm^−1^, attributed to C=O stretching of the methacrylate ester groups, and strong absorptions in the 1030–1100 cm^−1^ region correspond to P=O and P–O–C stretching vibrations from phosphate groups [54]. After PR dye adsorption on PVIB adsorbent (PVIBPR), new bands observed near 1410–1500 cm^−1^ can be assigned to aromatic C=C stretching and sulfonate (–SO_3_^−^) groups of PR dye, while the bands in the 620–700 cm^−1^ region correspond to C–S stretching [20]. These spectral modifications confirm the chemical interaction between the polymer network and PR dye, indicating successful incorporation of the dye into the PVIB matrix.

#### 3.1.2. Thermogravimetric Analysis (TGA)

The TGA curves of PVIB, PVIBPR, and PR (Figure 2b) provide insights into their thermal stability and decomposition patterns. Pure PR shows a slight weight loss below 150 °C, which is due to moisture evaporation, followed by a significant degradation step starting near 300 °C, corresponding to the breakdown of chromophoric structures and sulfonate groups in the dye [24]. For PVIB, an initial weight loss below 120–150 °C is attributed to the evaporation of physically adsorbed water and residual solvent. The primary decomposition occurs in the range of 250–450 °C, which corresponds to the degradation of the polymer backbone (vinyl imidazole and methacrylate units) and cleavage of phosphate linkages [55]. PVIBPR exhibits an earlier onset of degradation compared to PVIB, starting around 230 °C, which may result from the incorporation of PR dye, introducing additional weak points in the polymer matrix and lowering thermal stability [37,54]. However, PVIBPR retains a significant char residue above 500 °C, indicating the presence of thermally stable phosphate groups and aromatic dye fragments that contribute to char formation and structural integrity at high temperatures.

In accordance with the TGA results, the DTG profiles of (a) PVIB, (b) PVIBPR, and (c) PR further clarify their decomposition behavior (Figure S1). PR shows a main degradation peak around 300 °C, corresponding to the breakdown of its chromophoric and sulfonate groups. PVIB exhibits a two-step decomposition, with the first DTG peak attributed to the release of moisture or residual solvent and the second peak (250–450 °C) associated with the degradation of the polymer backbone. Incorporation of PR into the polymer matrix causes PVIBPR to begin degrading at a slightly lower temperature, confirming that the dye introduces more heat-sensitive sites and slightly reduces the initial thermal stability, as also observed in the TGA curve. Nevertheless, PVIBPR displays a broader high-temperature decomposition shoulder and a higher char yield, indicating that the phosphate units and aromatic dye fragments help form thermally stable carbonaceous residues at elevated temperatures.

#### 3.1.3. SEM-TEM Images and SEM-EDX Analysis

**Figure 3:**
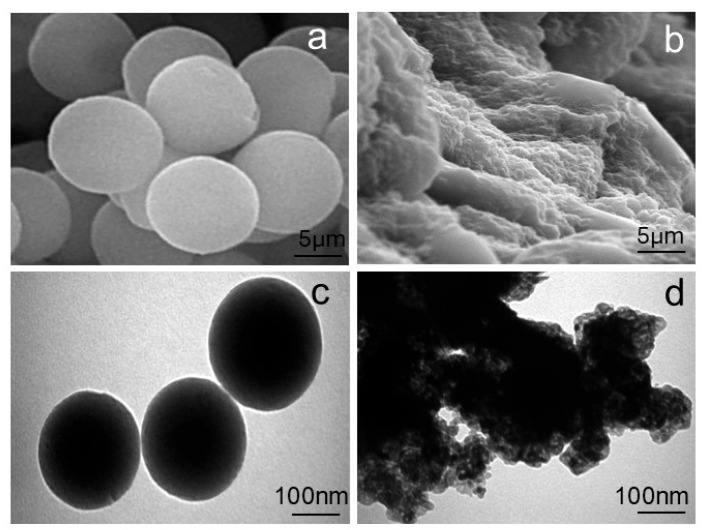
SEM image of (**a**) PVIB and (**b**) PVIBPR; TEM images of (**c**) PVIB and (**d**) PVIBPR.

The morphological characteristics of the PVIB adsorbent before and after PR dye adsorption were investigated using SEM and TEM, as presented in Figure 3a–d). The SEM image of the pristine PVIB (Figure 3a) reveals uniform, well-defined spherical particles with smooth surfaces and regular geometry, indicating a homogeneous polymeric structure. Complementary TEM analysis (Figure 3c) further confirms the spherical morphology with sharp boundaries and consistent size distribution in the nanometer range, reflecting a well-organized matrix with readily accessible adsorption sites. This morphology indicates successful polymerization and crosslinking of PVI with Bis[2-(methacryloyloxy)ethyl] phosphate, resulting in a stable and homogeneous polymeric network. Such morphological uniformity suggests high surface availability, which is advantageous for efficient dye uptake.

Following the dye adsorption, significant structural changes are observed. The SEM image of the dye-loaded PVIB (Figure 3b) shows roughened and irregular surfaces compared to the smooth texture of the virgin adsorbent. The spherical morphology appears to be partially masked, suggesting surface coverage by dye molecules and possible particle aggregation. Similarly, the TEM image (Figure 3d) reveals darker and less-defined structures, with evident agglomeration of particles. The disappearance of distinct spherical boundaries, along with the presence of dense regions, is indicative of strong dye adsorption and particle clustering. These morphological alterations can be attributed to surface modification and the occupation of active sites by dye molecules.

The observed changes are consistent with the proposed adsorption mechanism, in which electrostatic interactions between the imidazole groups of PVIB and the anionic sulfonate groups of the PR dye, along with possible hydrogen bonding, govern the strong binding. In summary, the transition from smooth, discrete particles in virgin PVIB to irregular, aggregated, and dye-coated structures in dye-loaded PVIB provides clear evidence of effective adsorption and validates the material’s suitability as an efficient adsorbent.

**Figure 4:**
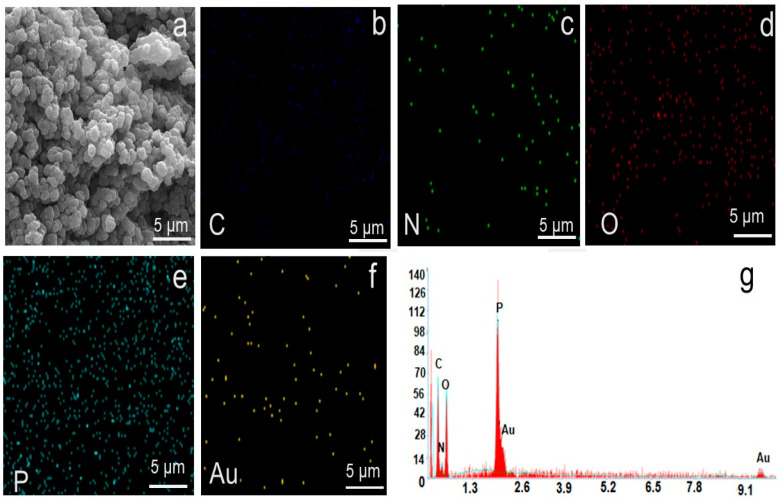
(**a**) SEM image of PVIB, (**b**) Carbon (C), (**c**) Nitrogen (N), (**d**) Oxygen (O), (**e**) Phosphorus (P), and (**f**) Gold (Au); (**g**) EDS mapping of PVIB elemental distribution obtained from EDS spectra.

The SEM-EDX analysis further supports the morphological changes observed in the SEM and TEM images of PVIB before and after PR dye adsorption. The virgin PVIB adsorbent exhibits a composition dominated by carbon (55.9%), nitrogen (14.5%), and oxygen (21.0%), consistent with the PVI backbone and phosphate incorporation. Phosphorus is detected at 0.7%, confirming the presence of Bis[2-(methacryloyloxy)ethyl] phosphate within the polymer matrix. The uniform elemental distribution seen in the EDX elemental mapping (Figure 4b–f) aligns with the smooth, spherical morphology observed in SEM and TEM images, suggesting a homogeneous structure with well-exposed active sites.

To further confirm the polymerization and elemental composition of the virgin PVIB, WD-XRF analysis was performed and compared with SEM-EDX results. The WD-XRF data yielded closely corresponding elemental mass percentages of C (60.7%), N (15.8%), O (22.8%), and P (0.7%) to those obtained from SEM-EDX analysis. The strong consistency between both analytical techniques reveals the successful incorporation of phosphate functional groups into the polymer matrix and confirms the compositional uniformity of the PVIB network. These results demonstrate that the polymerization of PVI with Bis[2-(methacryloyloxy)ethyl] phosphate proceeded effectively, producing a homogeneous polymeric structure consistent with the observed surface morphology.

**Figure 5:**
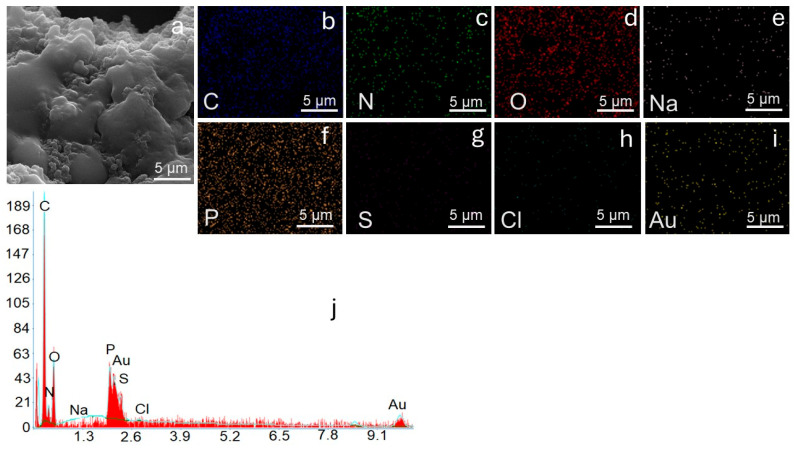
(**a**) SEM image of PVIBPR, (**b**) Carbon (C), (**c**) Nitrogen (N), (**d**) Oxygen (O), (**e**) Sodium (Na), (**f**) Phosphorus (P), (**g**) Sulphur (S), (**h**) Chloride (Cl), and (**i**) Gold (Au); (**j**) EDS mapping of PVIBPR Elemental distribution obtained from EDS spectra.

Elemental mapping in Figure 5b–i confirms the uniform presence of C and N from the polyvinyl imidazole backbone and O and P from the crosslinker, demonstrating the structural integrity of the polymer matrix after adsorption. After adsorption, significant compositional changes occur. The reduction in carbon (47.67%) and nitrogen (7.79%) alongside the pronounced increase in phosphorus (6.71%) suggests substantial surface modification and coverage by dye molecules. Notably, the appearance of sodium (1.52%), sulfur (0.76%), and chloride (0.57%) signals, absent in the virgin adsorbent, confirms the successful adsorption of PR dye, which contains sulfonate and chloride groups as characteristic functional moieties. In addition, detecting Na, S, and Cl distributed throughout the surface confirms the immobilization of sulfonated PR dye molecules. Au signals originate from the sputter-coated layer for imaging purposes. The EDX spectrum (Figure 5j) corroborates these findings, showing characteristic C, O, P, Na, S, and Cl peaks. The SEM-EDX analysis results agree well with SEM/TEM images, confirm that PVIB undergoes morphological transformation upon dye adsorption and achieves uniform distribution of the dye species within its structure.

#### 3.1.4. BET Results

BET analysis results of PVIB adsorbent before and after PR dye uptake are given in Table 2. The average pore radius decreased slightly from 5.56 to 5.41 Å, indicating partial pore filling by dye molecules. The multipoint BET surface area increased from 779 to 3654 m^2^/g, while BJH adsorption/desorption surface areas rose from ~430–450 to ~1900 m^2^/g. Similarly, pore volume expanded significantly, with total pore volume increasing from 0.217 to 0.989 cc/g.

Typically, adsorption processes reduce available surface area due to pore filling or blockage; however, the observed enhancement can be attributed to structural reorganization and swelling of the PVIB matrix, whereby dye–polymer interactions expose previously inaccessible pores. Despite these changes, BJH pore radii (~15.6 Å) remained stable, confirming mesoporous integrity. SEM and TEM analyses revealed that pristine PVIB consisted of uniform, spherical particles with smooth surfaces, while the dye-loaded PVIB displayed roughened, irregular surfaces and agglomerated morphologies, suggesting particle restructuring and surface modification. Possible interactions between the imidazole groups of PVIB and the sulfonated dye, such as electrostatic and hydrogen-bonding, likely promoted polymer swelling, partial fragmentation, and formation of new voids, thereby exposing additional internal surfaces and enhancing porosity. In several recent studies, researchers have observed increases in surface area or pore volume after dye (or pollutant) adsorption, consistent with our findings. For example, Qin et al. [56] reported that the specific surface area of modified biochar increased from 12.5 to 16.8 m^2^/g after phosphorus adsorption, attributed to pore re-distribution and micropore exposure. Agi et al. [57] found that the adsorption of reservoir fines by mesoporous silica nanoparticles increased the micropore volume from 0.011 to 0.019 cm^3^/g, mesopore volume from 0.029 to 0.041 cm^3^/g, and total pore volume from 0.040 to 0.060 cm^3^/g. Similarly, in the crosslinked diatomite–chitosan/calcium alginate composite, methylene blue uptake increased the BET surface area from 8.35 to 12.6 m^2^/g and the average pore size from 3.21 to 4.08 nm, indicating adsorption-induced exposure of new internal surfaces [58]. Overall, BET results of this study suggest strong interaction and high adsorption capacity of PVIB, its strong affinity for PR dye, and its promise as an efficient adsorbent for dye removal.

### 3.2. Effect of Adsorption Parameters

#### 3.2.1. Initial Solution pH

**Figure 6:**
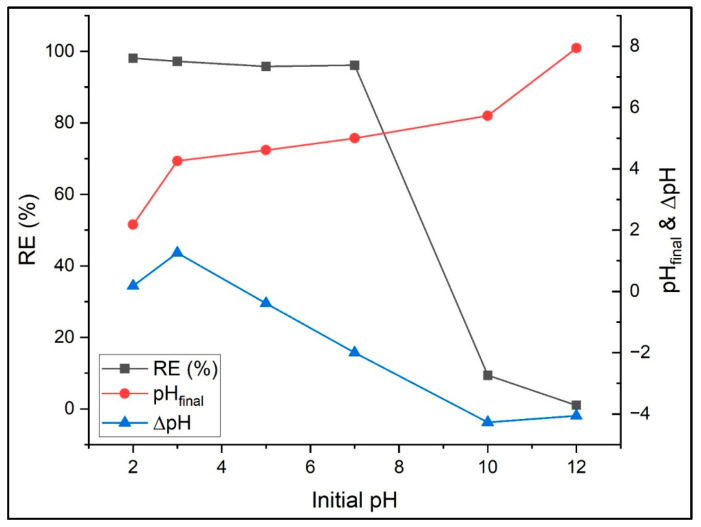
Effect of solution pH on removal efficiency.

The pH of the solution plays a crucial role in dye adsorption, as it directly influences the ionization state and surface charge of the functional groups present on the adsorbent. To evaluate this effect, the removal performance of PVIB adsorbents toward PR was examined across a wide pH range (2–12) (Figure 6). All other operating conditions were maintained constant throughout these experiments, with an adsorbent dosage of 75 mg, a contact time of 180 min, an initial dye concentration of 100 mg L^−1^, and a temperature of 25 °C.

The adsorption of PR onto PVIB shows strong dependence on solution pH due to changes in the surface charge of the adsorbent and the ionization state of the dye. At acidic conditions (pH 2–3), adsorption is highly efficient, with removal rates of 98.1% and 97.2%. This is attributed to the protonation of imidazole and phosphate groups on PVIB, forming a positively charged surface that strongly attracts the anionic dye through electrostatic forces. The small Δ_pH_ values suggest minimal proton exchange, though at pH 3 a slight increase indicates buffering or ion exchange during adsorption. In near-neutral conditions (pH 5–7), efficiency remains high (95.8–96.1%), although surface deprotonation reduces positive charge density. Strong adsorption persists, likely through hydrogen bonding and residual electrostatic interactions. The marked drop in final pH at pH 7 (Δ_pH_ = −2.0) indicates significant proton release, reflecting active surface–dye interactions. At alkaline pH (10–12), removal efficiency sharply declines to 9.4% and 1.0%. Under these conditions, imidazole and phosphate groups are largely deprotonated, giving the PVIB surface a negative charge. This generates strong electrostatic repulsion with the negatively charged dye, preventing adsorption despite proton release into solution (Δ_pH_ up to −4.3). Similar initial solution pH effect was reported in the literature for PR dye adsorption by homemade peach and commercial activated carbons, corncob activated carbon, and activated carbon-Fe_3_O_4_ composite adsorbents [19,20,27]. In summary, PVIB effectively removes PR dye molecules under acidic to neutral conditions, with electrostatic attraction as the primary mechanism, complemented by hydrogen bonding. However, adsorption is severely limited at alkaline pH due to surface charge repulsion, emphasizing the critical role of pH control in optimizing dye removal. From these findings, an initial solution pH of 7 was identified as the most favorable condition and was therefore selected for all subsequent experiments.

#### 3.2.2. Bis[2-(methacryloyloxy)ethyl] Phosphate Content

**Figure 7:**
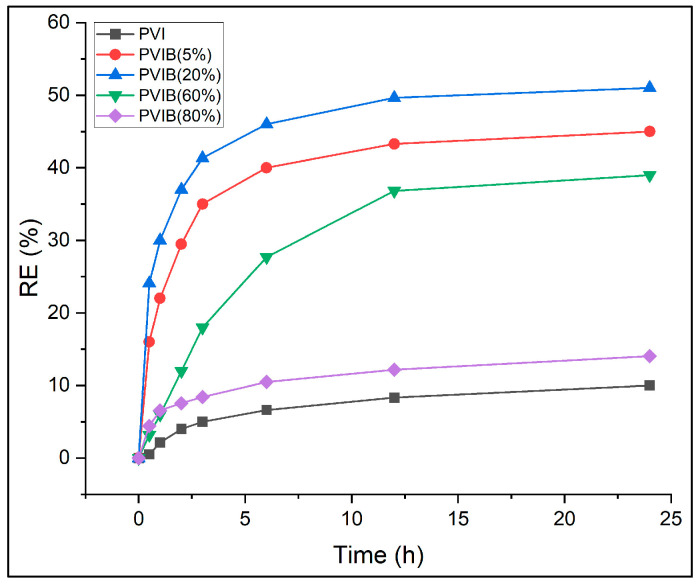
Effect of Bis[2-(methacryloyloxy)ethyl] phosphate content on removal efficiency.

The adsorption performance of PVI and PVI-based composite adsorbents modified with different loadings (5–80%) of bis[2-(methacryloyloxy)ethyl] phosphate (B) was investigated for the removal of PR dye. Batch adsorption tests were carried out using a fixed dye concentration of 100 mg L^−1^ and a uniform adsorbent mass of 20 mg. The tests were monitored for 24 h to evaluate time-dependent color removal efficiencies. Dye removal was quantified at several time intervals (0.5, 1, 2, 3, 6, 12, and 24 h) at 25 °C, and removal efficiencies of adsorbent formulations were compared in Figure 7.

The experimental results reveal that unmodified PVI exhibited limited adsorption capacity, with only 10% dye removal after 24 h. Incorporation of PVIB significantly enhanced performance, particularly at moderate loading levels. Among the composites, PVIB20% achieved the highest efficiency, removing 51% of dye after 24 h, outperforming both lower (5%) and higher (60–80%) B contents. PVIB5% showed gradual improvement, reaching 45% removal at equilibrium, whereas PVIB60% and PVIB80% were less effective, with 14.1% and 39% efficiencies, respectively.

The superior performance of PVIB20% can be attributed to an optimal balance between surface functionality and polymer matrix stability. The phosphate groups in PVIB likely enhanced electrostatic attraction between the negatively charged dye molecules and positively charged adsorption sites on the composite. At 20% B loading, sufficient active sites are available without causing structural crowding or reduced accessibility. In contrast, low B loading (5%) provides fewer binding sites, limiting dye uptake, while excessive loading (≥60%) may cause aggregation or blockage of pores, reduce effective surface area, and prevent mass transfer. Thus, the 20% PVIB composition achieves a synergistic effect and maximizes both surface interaction and porosity. From these findings, PVIB20% was identified as the best-performing adsorbent among the compositions studied and selected for all further experiments.

#### 3.2.3. Adsorbent Dose

**Figure 8:**
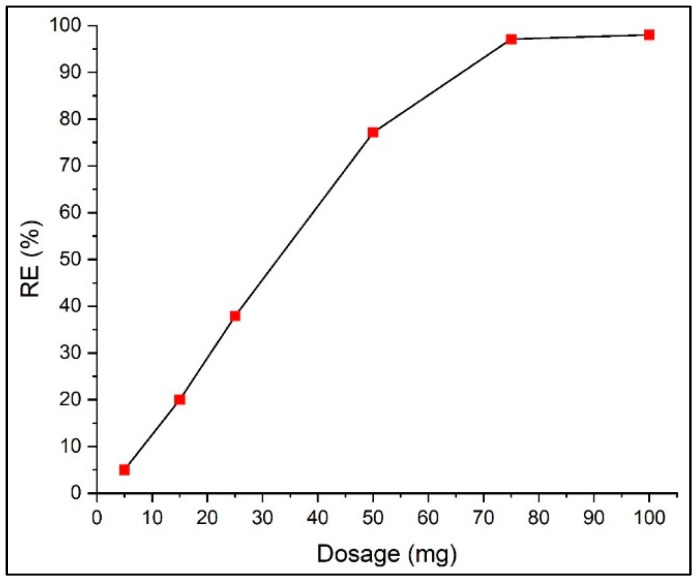
Effect of adsorbent dose on removal efficiency.

The effect of PVIB20% dosage on the adsorption of PR dye was investigated at an initial dye concentration of 100 mg L^−1^, 25 °C, and a contact time of 3 h, with dosages ranging from 5 to 100 mg. The results demonstrate a clear correlation between adsorbent dose and dye removal efficiency (Figure 8). At the lowest dose of 5 mg, only 5% of PR was removed, reflecting the insufficient number of available adsorption sites relative to dye molecules. Increasing the dosage to 15 and 25 mg enhanced the removal efficiency to 20% and 37.9%, respectively, due to the increased surface area and greater number of active imidazole and phosphate functional groups available for electrostatic interactions and hydrogen bonding with PR molecules. A more pronounced increase was observed at 50 mg, where removal efficiency reached 77.1%, indicating a significant improvement in dye–adsorbent interactions as surface sites became more abundant.

The removal efficiency approached saturation at above 50 mg of adsorbent dose. PR removal reached 97.1% at 75 mg, and a further increase to 100 mg improved efficiency only by 1%. This plateau effect can be attributed to the available adsorption sites exceeding the number of dye molecules present in solution at higher dosages, leading to incomplete adsorbent utilization. Similar behavior has been reported in dye adsorption studies using VI-based polymeric adsorbents, where excessive adsorbent loading offers no significant gain in removal but increases operational cost and material usage [35,36]. Therefore, 75 mg was selected as the optimum dose, as it achieves outstanding PR removal while maintaining cost-effectiveness and environmental sustainability.

#### 3.2.4. Contact Time and Initial Dye Concentration

Batch adsorption experiments were conducted to examine the influence of contact time and initial dye concentration on PR dye removal using PVIB20% adsorbent. A constant adsorbent dose of 75 mg was added to dye solutions with initial concentrations of 50, 75, 100, 250, and 500 mg L^−1^ at 25 °C. The suspensions were agitated, and aliquots were withdrawn at predetermined contact times ranging between 10 and 180 min. Residual dye concentrations were analyzed to determine removal efficiencies. The results reveal that both contact time and initial dye concentration significantly affect the adsorption process.

**Figure 9:**
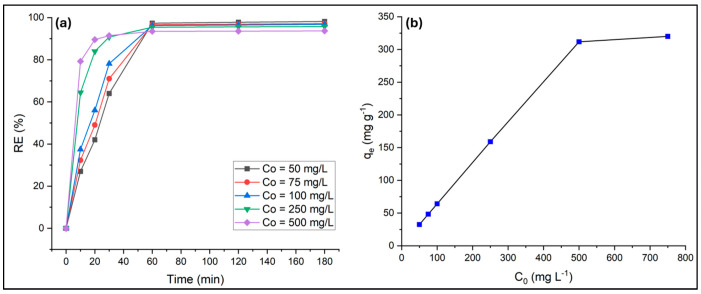
Effect of contact time (**a**) and initial dye concentration (**b**) on PR adsorption.

At low concentrations (50–100 mg L^−1^), removal efficiency increased steadily in the first 30 min (64–78%) and reached near-complete removal (96–98%) within 60 min (Figure 9a). This demonstrates that the available active sites on PVIB20% were sufficient to capture almost all dye molecules at lower loadings. At higher concentrations (250 and 500 mg L^−1^), adsorption was initially rapid, achieving over 90% removal within 30 min. However, equilibrium was reached at slightly lower efficiencies (93–96%) compared to dilute solutions. This reduction is explained by competition among excess dye molecules for a limited number of active sites, leaving some dye unabsorbed even at extended contact times. The sharp rise in removal efficiency during the early stages indicates fast surface adsorption, likely driven by strong electrostatic interactions between the negatively charged phosphate groups of the dye and the positively charged imidazole groups of PVIB20%. Beyond this stage, slower intraparticle diffusion or site saturation possibly limited further uptake. An optimized contact time of 60 min is selected for further experiments, since removal efficiencies across all initial concentrations already reach ~93.5–97.4% by 60 min and change by less than ~1% thereafter.

The equilibrium adsorption capacity (q_e_) of the adsorbent at 60 min of contact time increased significantly with rising initial dye concentration (C_0_), reflecting strong adsorption affinity and effective utilization of active sites (Figure 9b). A progressive increase in the q_e_ was observed up to 500 mg L^−1^, where the maximum capacity reached 312 mg g^−1^, attributed to the abundant availability of binding sites and the enhanced mass transfer driving force at higher concentrations. However, the q_e_ exhibited only a slight increase of 8 mg g^−1^ at 750 mg L^−1^ due to surface site saturation and limited additional uptake capacity.

### 3.3. Adsorption Kinetics

**Figure 10:**
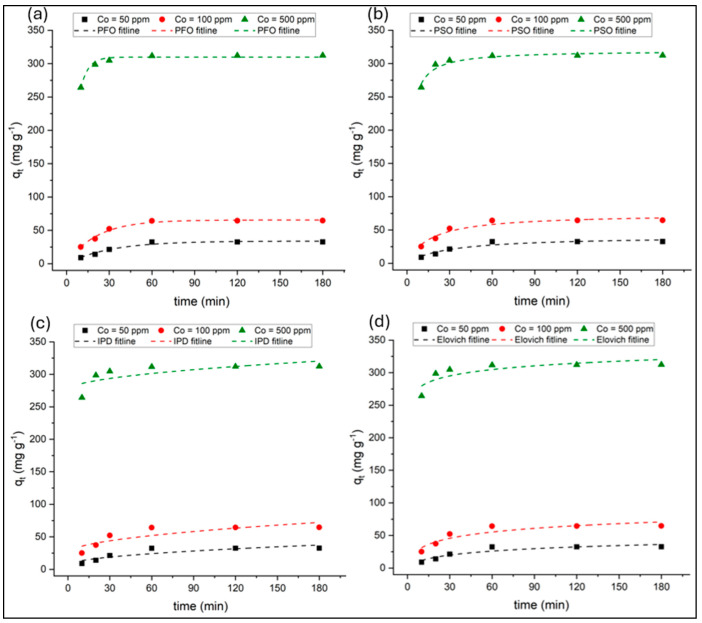
Fits of kinetic models to the experimental data (**a**): pseudo-first-order, (**b**): pseudo-second-order, (**c**): intraparticle diffusion, and (**d**): Elovich.

Adsorption kinetics were systematically evaluated using non-linear regression to ensure statistically robust parameter estimation at three initial dye concentrations (C_0_ = 50, 100, and 500 mg L^−1^) over contact times ranging from 10 to 180 min at 25 °C. The kinetic parameters and model fit metrics for the pseudo-first-order (PFO), pseudo-second-order (PSO), intraparticle diffusion (IPD), and Elovich models were determined for their respective standard equations and graphical plots, as outlined in Table 1. The calculated values are summarized in Table 3, while the standard model fits to the experimental data are presented in Figure 10.

Among the tested kinetic models, both the PFO and PSO models provided strong agreement with the experimental data, but their statistical performance varied depending on the dye concentration. For the PFO model, the calculated equilibrium capacities (q_e,cal_) were close to the experimental values (q_e,exp_), and Adj-R^2^ values ranged from 0.959 to 0.981, with reduced χ^2^ values between 4.51 and 12.2. The corresponding AIC and BIC values (24.6–30.6 and 12.0–18.0, respectively) were the lowest among all models, indicating that the PFO model most accurately represented the adsorption kinetics. These results suggest that the adsorption process is controlled by surface reaction kinetics with significant physical adsorption contributions.

In contrast, the PSO model, while traditionally indicative of chemisorption, exhibited slightly lower statistical agreement (Adj-R^2^ = 0.910–0.923) and higher χ^2^, AIC, and BIC values than PFO model. This implies that the adsorption of PR dye onto PVIB20% may not be governed solely by valence or electron-sharing mechanisms but rather involves combined physisorption and surface interaction processes. Nevertheless, the PSO model’s relatively high Adj-R^2^ values confirm that chemisorption still contributes significantly to the overall mechanism.

The IPD and Elovich models yielded comparatively poorer fits, characterized by higher χ^2^ and AIC/BIC values, particularly at higher dye concentrations. The IPD model was applied to assess whether diffusion governs the adsorption of PR dye onto PVIB20% adsorbent. For the IPD model, Adj-R^2^ decreased from 0.720 to 0.398 with increasing C_0_, indicating that intraparticle diffusion alone does not govern the rate-limiting step. The C values, which increased substantially at higher dye loadings, further suggest the presence of a boundary layer effect. The multilinear plots revealed two distinct stages for the IPD model. The k_d_, C, and R^2^ values of IPD model for these two stages can be found in Supplementary Table S1. The first stage, characterized by higher diffusion rate constants (k_d1_), corresponds to external mass transfer where PR molecules rapidly move from solution to the adsorbent surface. The second stage, with lower k_d2_ values, represents slower intraparticle diffusion into pores and active sites. Increasing initial dye concentration enhanced k_d1_, confirming stronger driving forces for external diffusion at higher gradients. However, as none of the lines passed through the origin, intraparticle diffusion was not the sole rate-limiting step.

The Elovich model is particularly suitable for describing adsorption processes occurring on energetically heterogeneous surfaces, where the activation energy of chemisorption varies with surface coverage [59]. The Elovich model indicated moderate fits (Adj-R^2^ = 0.615–0.845) with relatively high χ^2^ and AIC values, implying that surface heterogeneity and varying activation energies play secondary roles in the adsorption process. The adsorption rate constant (α) increased markedly with increasing initial dye concentration, indicating an enhanced initial adsorption rate at higher concentrations. The desorption constant (β) showed a noticeable decrease from 50 ppm to 100 ppm, followed by minimal change at higher concentration (500 ppm), suggesting a relatively stable desorption tendency at elevated dye concentrations.

Overall, the non-linear regression analysis confirms that the PFO model provides the best overall description of PR dye adsorption onto PVIB20%, supported by its lowest χ^2^, AIC, and BIC values and highest Adj-R^2^. These results emphasize that the process is predominantly surface-controlled, involving rapid physical interactions followed by slower chemical adsorption and intraparticle diffusion contributions during the later stages.

### 3.4. Adsorption Isotherms

**Figure 11:**
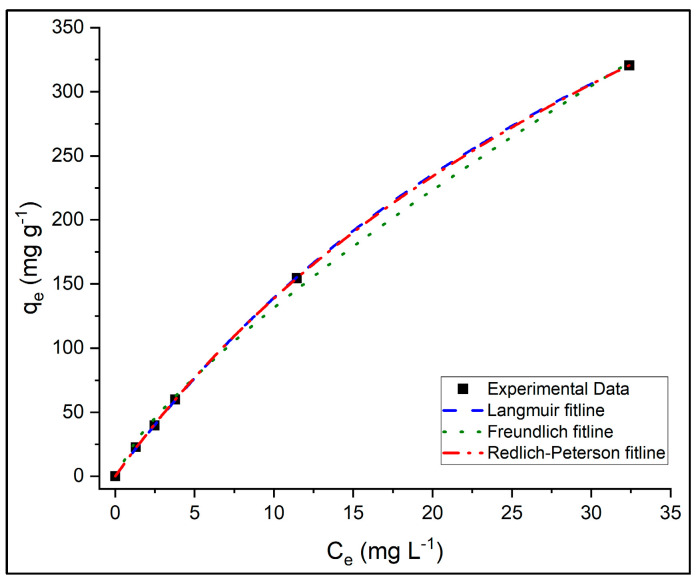
The adsorption isotherms of PR dye on PVIB20% adsorbent.

The adsorption behavior of PR dye on PVIB20% was analyzed using the standard forms of the Langmuir, Freundlich, and Redlich–Peterson (R–P) isotherm models. Model parameters were determined through non-linear regression in OriginPro software, employing the Levenberg–Marquardt algorithm, which preserves the original error structure and ensures statistically consistent parameter estimation. The corresponding isotherm model parameters and fit statistics are summarized in Table 4. Figure 11 shows the fitted adsorption isotherms obtained at initial dye concentrations of 50, 75, 100, 250, 500 mg L^−1^. The x-axis represents the equilibrium concentration of PR dye in solution (C_e_), and the y-axis represents the equilibrium adsorption capacity (q_e_). Experimental data are compared with Langmuir, Freundlich, and Redlich–Peterson model fits.

The Langmuir model effectively described the adsorption process with Adj-R^2^ = 0.9999, reduced-χ^2^ = 0.936, AIC = 27.1, and BIC = 1.94, and a low MPSD value (2.99), indicating a very good agreement between experimental and predicted equilibrium data. The model yielded a maximum monolayer adsorption capacity (q_m_) of 765 mg g^−1^ and a Langmuir constant (K_L_ × 10^−3^) of 22.3 L mg^−1^, demonstrating a strong affinity between PR dye molecules and the PVIB20% surface. The dimensionless separation factor (R_L_) values, shown in Supplementary Figure S2, all fall within the range of 0–1, confirming favorable adsorption behavior. Moreover, the logarithmic decrease in R_L_ with increasing C_0_ indicates enhanced adsorption favorability and partial irreversibility at higher dye concentrations, consistent with a high-affinity monolayer adsorption mechanism.

The Freundlich model also provided a reasonable fit (Adj-R^2^ = 0.9969) but showed higher reduced-χ^2^ (47.0), AIC (46.7), BIC (21.5), and MPSD (12.8) values compared to Langmuir and R–P models. The Freundlich constants—K_F_ = 22.5 mg g^−1^ and 1/n = 0.769—indicate favorable adsorption (0 < 1/n < 1), reflecting surface heterogeneity arising from the presence of phosphate functional groups and imidazole moieties. Although the Freundlich model accurately represents adsorption on heterogeneous surfaces, its relatively higher error metrics suggest that it does not fully capture the monolayer adsorption behavior dominating PR uptake on PVIB20%.

Among the three models, the R–P isotherm exhibited the best overall statistical performance, characterized by the lowest MPSD (2.09), reduced-χ^2^ (0.658), and AIC (25.5) values, while maintaining the same Adj-R^2^ (0.9999) as the Langmuir model. The BIC value (2.15) was slightly higher than that of Langmuir (1.94), reflecting the model’s inclusion of an additional fitting parameter. The obtained parameters—K_rp_ = 17.6 L g^−1^, α_rp_ × 10^−3^ = 34.3 L mg^−1^, and β = 0.899—indicate that the adsorption mechanism predominantly follows a Langmuir-type monolayer process (since β approaches 1) with minor contributions from surface heterogeneity. This model effectively bridges the gap between ideal homogeneous adsorption and realistic surface irregularities, yielding a more precise statistical representation of the experimental data.

Overall, while the Langmuir model provides clear physical understanding into monolayer adsorption on uniform surfaces, the R–P model statistically offers the most accurate fit to the experimental data, as supported by its lowest reduced-χ^2^, AIC, and MPSD values. The consistency of both models highlights that PR adsorption onto PVIB20% is highly favorable, predominantly monolayer in nature, and occurs on a largely homogeneous surface with slight heterogeneity effects. The high adsorption capacity of 765 mg g^−1^ confirms PVIB20% as an efficient and stable adsorbent for anionic dye removal.

### 3.5. Adsorption Thermodynamics

**Figure 12:**
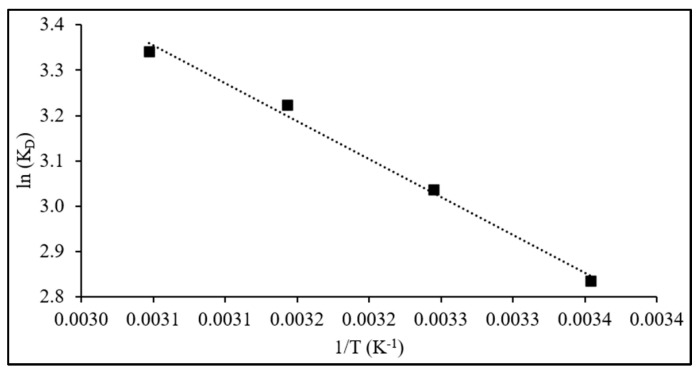
Linear plot of ln(K_D_) versus 1/T for PR adsorption on PVIB20%.

The thermodynamic parameters, including K_D_, ΔH^0^, and ΔS^0^, and ΔG^0^, were computed by applying Equations (14) and (15) to the adsorption data obtained at temperatures 299, 308, 318, and 328 K, initial dye concentration of 100 mg L^−1^, 60 min of contact time, and 75 mg of adsorbent dose, and results are given in Table 5. The PVIB system shows high removal across all temperatures (96.2–97.7%), with a modest increase as temperature rises, indicating thermally assisted uptake (Figure 12).

Negative ΔG^0^ values (−7.05 to −9.15 kJ mol^−1^) confirm spontaneous adsorption, with more negative values at higher temperature indicating enhanced feasibility. While these values lie in the borderline range between physisorption and chemisorption, their temperature dependence suggests progressively stronger surface interactions. The positive ΔH^0^ (13.9 kJ mol^−1^) reveals an endothermic process. Although modest, this enthalpy magnitude is consistent with chemisorption involving multiple weak-to-moderate forces—electrostatic attraction, hydrogen bonding, and π–π/heteroatom interactions—rather than van der Waals-driven physisorption. The positive ΔS^0^ (70 J mol^−1^ K^−1^) indicates increased randomness at the interface, arising from desolvation and structural rearrangements as dye molecules associate with imidazole and phosphate groups.

Comparable studies have shown ΔH^0^ values of 10–40 kJ mol^−1^ for chemisorption, whereas values below 10 kJ mol^−1^ are typical of weak physisorption. Likewise, ΔG^0^ values in the −5 to −20 kJ mol^−1^ range have been attributed to spontaneous chemisorption in dye–polymer systems. The present results (ΔH^0^ = 13.9 kJ mol^−1^; ΔG^0^ = −7.05 to −9.15 kJ mol^−1^) fall within these reported ranges, reinforcing a chemisorption-driven mechanism [3,60,61]. The kinetic, isotherm, and thermodynamic analyses collectively confirm that PR dye adsorption onto PVIB20% is a spontaneous, endothermic, and surface-controlled process. Adsorption occurs predominantly as monolayer coverage via rapid physisorption followed by weak chemisorptive interactions on homogeneous active sites, ensuring high affinity and thermal enhancement of dye uptake.

### 3.6. Literature Comparison

A comparative summary of PR dye adsorption studies reported in the literature, including maximum adsorption capacity (q_max_), initial dye concentration (C_0_), equilibrium time (t_e_), and corresponding kinetic, isotherm, and thermodynamic behaviors, is presented in Table 6. This comprehensive comparison highlights both the quantitative and mechanistic distinctions among various adsorbents.

Conventional materials such as corncob activated carbon (2.86 mg g^−1^, [19]), modified bentonite (8.50 mg g^−1^, [23]), and alumina–activated carbon composites (12.4 mg g^−1^, [22]) exhibit low adsorption capacities, primarily following first- or second-order kinetics with Langmuir-type isotherms but limited thermal stability. In contrast, more advanced systems such as magnetite–humic acid MgAl layered double hydroxide (192 mg g^−1^, [26]) and HNO_3_-treated avocado shells (213 mg g^−1^, [21]) show endothermic or exothermic adsorption, depending on surface chemistry, and are better described by Sips or generalized order models (GOM), though they still require longer equilibrium times (90–180 min).

Carbon-based composites like activated carbon–Fe_3_O_4_ (278 mg g^−1^, [27]) and activated peach carbon (297 mg g^−1^, [20]) fit PFO or Freundlich/Liu isotherms, reflecting heterogeneous multilayer adsorption with slower kinetics. Similarly, modified montmorillonite (319 mg g^−1^, [24]) exhibits endothermic adsorption governed by the Liu model, but equilibrium is reached only after 430 min, indicating diffusion limitations.

In contrast, the PVIB adsorbent developed in this study exhibits an exceptional q_max_ of 765 mg g^−1^ at C_0_ = 500 mg L^−1^, attaining equilibrium within 60 min. The PVIB outperforms previously reported adsorbents not only in adsorption capacity but also in kinetic efficiency, equilibrium behavior, thermodynamic favorability, and equilibrium time. Its rapid, monolayer, and thermally promoted adsorption mechanism establishes PVIB as a highly efficient and mechanistically well-understood adsorbent for the removal of anionic dyes from wastewater.

## 4. Conclusions

This study presents the design, synthesis, and application of novel Poly(1-vinyl imidazole)-Bis[2-(methacryloyloxy)ethyl] phosphate (PVIB) polymers as efficient adsorbents for textile dye removal. Through systematic characterization, PVIB was shown to exhibit well-defined spherical morphology, tunable surface chemistry, and high structural stability. The incorporation of phosphate groups not only enhanced electrostatic interactions with anionic dyes but also provided robust frameworks for adsorption. Among different formulations, PVIB20% demonstrated the best balance of functionality and stability, achieving an exceptional equilibrium adsorption capacity of 330 mg g^−1^ for Procion Red dye within 60 min.

Comprehensive kinetic, equilibrium, and thermodynamic analyses demonstrated that Procion Red dye adsorption onto PVIB20% is a spontaneous, endothermic, and surface-controlled process. The adsorption kinetic modeling results identified the pseudo-first-order model as the most suitable descriptor, indicating that the process is governed primarily by rapid physisorption, followed by weaker chemisorptive interactions and limited intraparticle diffusion. The adsorption isotherm analysis revealed that adsorption occurs predominantly as a monolayer process on a largely homogeneous surface, with the Langmuir and Redlich–Peterson models showing the best statistical agreement. The Langmuir model estimated a maximum monolayer adsorption capacity (q_m_) of 765 mg·g^−1^, confirming the high affinity and efficient utilization of available active sites. Thermodynamic parameters indicate a spontaneous, endothermic, and entropy-driven adsorption mechanism, consistent with electrostatic and hydrogen-bonding interactions between PR dye molecules and the imidazole–phosphate functional sites of PVIB20%.

In comparison with reported adsorbents, PVIB20% exhibited an outstanding adsorption capacity and rapid equilibrium within 60 min, outperforming most conventional and modified materials in both efficiency and kinetic response. These results demonstrate that the adsorption behavior of PVIB20% is mechanistically consistent, experimentally validated, and theoretically sound, establishing it as a promising and high-capacity adsorbent for efficient removal of anionic dyes from aqueous systems.

## Figures and Tables

**Table 2:** BET results of PVIB adsorbent before and after PR dye uptake.

BET Parameters	PVIB	PVIB After PR Adsorption
Average pore Radius (Å)	5.56	5.41
BJH adsorption Surface Area (m^2^/g)	428	1944
BJH adsorption Pore Volume (cc/g)	0.584	2.64
BJH adsorption Pore Radius Dv(r) (Å)	15.7	15.6
BJH desorption Surface Area (m^2^/g)	449	1916
BJH desorption Pore Volume (cc/g)	0.578	2.61
BJH desorption Pore Radius Dv(r) (Å)	15.6	15.6
Multipoint BET surface area (m^2^/g)	779	3654
Total pore volume (cc/g)	0.217	0.989

**Table 3:** Parameters, experimental $q_{e}$, and model fit metrics for kinetic models.

Model	Parameter	C_0_ = 50 ppm	C_0_ = 100 ppm	C_0_ = 500 ppm
Experimental	q_e,exp_	32.7	64.5	312
Pseudo-First-Order	q_e,cal_	33.9	65.5	310
	k_1_	0.033	0.048	0.189
	Adj-R^2^	0.959	0.981	0.965
	Reduced χ^2^	4.51	5.28	12.2
	AIC	24.6	25.5	30.6
	BIC	12.0	12.9	18.0
Pseudo-Second-Order	q_e,cal_	40.7	74.4	320
	k_2_ × 10^−3^	0.851	0.812	1.67
	Adj-R^2^	0.910	0.923	0.923
	Reduced χ^2^	9.99	21.4	26.8
	AIC	29.4	34.0	35.3
	BIC	16.8	21.3	22.7
Intraparticle Diffusion	k_d_	2.33	3.56	3.37
	C	6.12	24.4	275
	Adj-R^2^	0.720	0.645	0.398
	Reduced χ^2^	31.0	99.2	209
	AIC	36.2	43.2	47.6
	BIC	23.5	30.5	35.0
Elovich	α	2.21	11.2	6.01 × 10^8^
	β × 10^−3^	103	70.1	71.1
	Adj-R^2^	0.845	0.817	0.615
	Reduced χ^2^	17.2	51.2	133
	AIC	32.6	39.2	44.92
	BIC	20.0	26.6	32.3

**Table 4:** Comparison of standard isotherm model parameters and fit statistics for PR adsorption on PVIB20%.

Isotherm Models			Model Fit Statistics				
Model	Parameter	Value	MPSD	Adj-R^2^	Reduced χ^2^	AIC	BIC
Langmuir	q_m_	765	2.99	0.9999	0.936	27.1	1.94
	K_L_ × 10^−3^	22.3					
Freundlich	K_F_	22.5	12.8	0.9969	47.0	46.7	21.5
	1/n	0.769					
Redlich–Peterson	K_rp_	17.6	2.09	0.9999	0.658	25.5	2.15
	α_rp_ × 10^−3^	34.3					
	β	0.899					

**Table 5:** Thermodynamic parameters’ values of PR adsorption on PVIB20%.

T (K)	ΔG^0^ (kJ mol^−1^)	ΔH^0^ (kJ mol^−1^)	ΔS^0^ (J mol^−1^ K^−1^)
298	−7.05	13.9	70
308	−7.75		
318	−8.45		
328	−9.15		

**Table 6:** Comparison of PR dye adsorption capacities of various adsorbents.

Adsorbent	q_max_ (mg g^−1^)	C_0_ (mg L^−1^)	t_e_ (min)	Kinetics	Isotherms	Thermo Dynamics	Reference
Corncob activated carbon	2.86	20	240	PSO	Langmuir	-	[19]
Modified bentonite	8.50	60	30	PFO	Langmuir	Exothermic	[23]
Alumina–activated carbon composite	12.4	30	120	PFO	-	-	[22]
Synthetic talc	12.9	60	960	-	Langmuir	-	[25]
Magnetite humic acid-decorated MgAl layered double hydroxide	192	200	180	PSO	Langmuir	Endothermic	[26]
HNO_3_-treated avocado shells	213	300	90	GOM	Sips	Exothermic	[21]
Activated carbon-Fe_3_O_4_ composite	278	150	50	PFO	Freundlich	-	[27]
Activated peach carbon	297	500	120	GOM	Liu model	Exothermic	[20]
Modified montmorillonite	319	300	430	-	Liu model	Endothermic	[24]
Poly (1-vinyl imidazole)-Bis[2-(methacryloyloxy)ethyl] Phosphate (PVIB)	765	500	60	PFO	Langmuir	Endothermic	This study

## Data Availability

The raw data supporting the conclusions of this article will be made available by the authors on request.

## Supplementary Materials

The following supporting information can be downloaded at: https://www.mdpi.com/article/10.3390/nano15221708/s1, Figure S1: TGA/DTG graph of (a) PVIB, (b) PVIBPR, (c) PR; Figure S2: The change in RL value within the studied C_0_ range; Table S1: Parameters and R^2^ values of two-step plots of IPD model.

## References

[1] Lin J., Ye W., Xie M., Seo D.H., Luo J., Wan Y., Van Der Bruggen B. (2023). Environmental Impacts and Remediation of Dye-Containing Wastewater. Nat. Rev. Earth Environ..

[2] Al-Tohamy R., Ali S.S., Li F., Okasha K.M., Mahmoud Y.A.-G., Elsamahy T., Jiao H., Fu Y., Sun J. (2022). A Critical Review on the Treatment of Dye-Containing Wastewater: Ecotoxicological and Health Concerns of Textile Dyes and Possible Remediation Approaches for Environmental Safety. Ecotoxicol. Environ. Saf..

[3] Sudarsan S., Murugesan G., Varadavenkatesan T., Vinayagam R., Selvaraj R. (2025). Efficient Adsorptive Removal of Congo Red Dye Using Activated Carbon Derived from Spathodea Campanulata Flowers. Sci. Rep..

[4] Yaseen D.A., Scholz M. (2019). Textile Dye Wastewater Characteristics and Constituents of Synthetic Effluents: A Critical Review. Int. J. Environ. Sci. Technol..

[5] Dutta P., Rabbi M.R., Sufian M.A., Mahjebin S. (2022). Effects of Textile Dyeing Effluent on the Environment and Its Treatment: A Review. Eng. Appl. Sci. Lett..

[6] Hoque M.B., Oyshi T.H., Hannan M.A., Haque P., Rahman M.M., Shahid M.A., Sheikh S. (2024). Unraveling the Ecological Footprint of Textile Dyes: A Growing Environmental Concern. Pollut. Stud..

[7] Adesanmi B.M., Hung Y.-T., Paul H.H., Huhnke C.R. (2022). Comparison of Dye Wastewater Treatment Methods: A Review. GSC Adv. Res. Rev..

[8] Tabish M., Tabinda A.B., Mazhar Z., Yasar A., Ansar J., Wasif I. (2024). Physical, Chemical and Biological Treatment of Textile Wastewater for Removal of Dyes and Heavy Metals. Desalination Water Treat..

[9] Bal G., Thakur A. (2022). Distinct Approaches of Removal of Dyes from Wastewater: A Review. Mater. Today Proc..

[10] Asghar A., Abdul Raman A.A., Wan Daud W.M.A. (2015). Advanced Oxidation Processes for In-Situ Production of Hydrogen Peroxide/Hydroxyl Radical for Textile Wastewater Treatment: A Review. J. Clean. Prod..

[11] Khader E.H., Muslim S.A., Saady N.M.C., Ali N.S., Salih I.K., Mohammed T.J., Albayati T.M., Zendehboudi S. (2024). Recent Advances in Photocatalytic Advanced Oxidation Processes for Organic Compound Degradation: A Review. Desalination Water Treat..

[12] Ziembowicz S., Kida M. (2025). The Optimization of Advanced Oxidation Processes for the Degradation of Industrial Pollutants. Sustainability.

[13] Bhatia D., Sharma N.R., Singh J., Kanwar R.S. (2017). Biological Methods for Textile Dye Removal from Wastewater: A Review. Crit. Rev. Environ. Sci. Technol..

[14] Ledakowicz S., Paździor K. (2021). Recent Achievements in Dyes Removal Focused on Advanced Oxidation Processes Integrated with Biological Methods. Molecules.

[15] Agarwala R., Mulky L. (2023). Adsorption of Dyes from Wastewater: A Comprehensive Review. ChemBioEng Rev..

[16] Anil I., Gunday S.T., Bozkurt A., Alagha O. (2020). Design of Crosslinked Hydrogels Comprising Poly(Vinylphosphonic Acid) and Bis [2-(Methacryloyloxy)Ethyl] Phosphate as an Efficient Adsorbent for Wastewater Dye Removal. Nanomaterials.

[17] Kandisa R.V., Saibaba Kv N., Shaik K.B., Gopinath R. (2016). Dye Removal by Adsorption: A Review. J. Bioremediat. Biodegrad..

[18] Kassa A., Engida A., Endaye M. (2025). Eco-Friendly Adsorbents for Industrial Dye Removal: A Comprehensive Review of Low-Cost Alternatives. Desalination Water Treat..

[19] Nazifa T.H., Habba N., Salmiati, Aris A., Hadibarata T. (2018). Adsorption of Procion Red MX-5B and Crystal Violet Dyes from Aqueous Solution onto Corncob Activated Carbon. J. Chin. Chem. Soc..

[20] Ribas M.C., Franco M.A.E.D., Adebayo M.A., Lima E.C., Parkes G.M.B., Feris L.A. (2020). Adsorption of Procion Red MX-5B Dye from Aqueous Solution Using Homemade Peach and Commercial Activated Carbons. Appl. Water Sci..

[21] Georgin J., da Silva Marques B., da Silveira Salla J., Foletto E.L., Allasia D., Dotto G.L. (2018). Removal of Procion Red Dye from Colored Effluents Using H2SO4-/HNO3-Treated Avocado Shells (*Persea americana*) as Adsorbent. Environ. Sci. Pollut. Res..

[22] Fatma F., Hariani P.L., Riyanti F., Sepriani W. (2018). Desorption and Re-Adsorption of Procion Red MX-5B Dye on Alumina-Activated Carbon Composite. Indones. J. Chem..

[23] Iqajtaoune A., Taibi M., Saufi H., Aouan B., Boudad L. (2024). Enhanced Removal of Methylene Blue and Procion Deep Red H-EXL Dyes from Aqueous Environments by Modified-Bentonite: Isotherm, Kinetic, and Thermodynamic. Desalination Water Treat..

[24] Thue P.S., Teixeira R.A., Hounfodji J.W., Machado F.M., Mello B.L., Andreazza R., Naushad M., Dehmani Y., Badawi M., Lima E.C. (2024). Intercalation of Organosilane in Clay Mineral for the Removal of Procion Red MX-5B: Investigational and Theoretical Studies. Sep. Purif. Technol..

[25] Rahman A., Urabe T., Kishimoto N. (2013). Color Removal of Reactive Procion Dyes by Clay Adsorbents. Procedia Environ. Sci..

[26] Ahmad N., Rohmatullaili, Wijaya A., Lesbani A. (2024). Magnetite Humic Acid-Decorated MgAl Layered Double Hydroxide and Its Application in Procion Red Adsorption. Colloids Surf. A Physicochem. Eng. Asp..

[27] Hariani P.L., Faizal M., Ridwan, Marsi, Setiabudidaya D. (2018). Removal of Procion Red MX-5B from Songket’s Industrial Wastewater in South Sumatra Indonesia Using Activated Carbon-Fe3O4 Composite. Sustain. Environ. Res..

[28] Sahiner N., Ozay O. (2011). Highly Charged p(4-Vinylpyridine-Co-Vinylimidazole) Particles for Versatile Applications: Biomedical, Catalysis and Environmental. React. Funct. Polym..

[29] Sun J., Jin Z., Wang J., Wang H., Zhang Q., Gao H., Jin Z., Zhang J., Wang Z. (2023). Application of Ionic Liquid Crosslinked Hydrogel for Removing Heavy Metal Ions from Water: Different Concentration Ranges with Different Adsorption Mechanisms. Polymers.

[30] Hezarkhani M., Ustürk S., Özbilenler C., Yilmaz E. (2024). pH-Responsive Pullulan Based Adsorbent Functionalized by Poly(N-Vinylimidazole): Synthesis, Characterization and Dye Removal Application. J. Water Chem. Technol..

[31] Abu Elella M.H., Goda E.S., Abdallah H.M., Shalan A.E., Gamal H., Yoon K.R. (2021). Innovative Bactericidal Adsorbents Containing Modified Xanthan Gum/Montmorillonite Nanocomposites for Wastewater Treatment. Int. J. Biol. Macromol..

[32] Banerjee P., Dinda P., Kar M., Uchman M., Mandal T.K. (2023). Ionic Liquid Cross-Linked High-Absorbent Polymer Hydrogels: Kinetics of Swelling and Dye Adsorption. Langmuir.

[33] Hingrajiya R.D., Patel M.P. (2023). Fe_3_O_4_ Modified Chitosan Based Co-Polymeric Magnetic Composite Hydrogel: Synthesis, Characterization and Evaluation for the Removal of Methylene Blue from Aqueous Solutions. Int. J. Biol. Macromol..

[34] Hu F., Fang C., Wang Z., Liu C., Zhu B., Zhu L. (2017). Poly (N-Vinyl Imidazole) Gel Composite Porous Membranes for Rapid Separation of Dyes through Permeating Adsorption. Sep. Purif. Technol..

[35] Kıvanç M.R., Ozay O., Ozay H., Ilgin P. (2022). Removal of Anionic Dyes from Aqueous Media by Using a Novel High Positively Charged Hydrogel with High Capacity. J. Dispers. Sci. Technol..

[36] Bildik F., Turan G.T., Duran H., Şişmanoğlu T., Senkal B.F. (2019). Sorption of Acidic Dyes from Water by Poly(Vinyl Imidazole) Grafted onto Poly(Styrene) Based Beads. Desalination Water Treat..

[37] Şenel M., Coşkun A., Fatih Abasıyanık M., Bozkurt A. (2010). Immobilization of Urease in Poly(1-Vinyl Imidazole)/Poly(Acrylic Acid) Network. Chem. Pap..

[38] Benjelloun M., Miyah Y., Akdemir Evrendilek G., Zerrouq F., Lairini S. (2021). Recent Advances in Adsorption Kinetic Models: Their Application to Dye Types. Arab. J. Chem..

[39] Dąbrowski A. (2001). Adsorption—From Theory to Practice. Adv. Colloid Interface Sci..

[40] Lagergren S. (1898). Zur Theorie Der Sogenannten Adsorption Geloster Stoffe, Kungliga Svenska Vetenskapsakademiens. Handlingar.

[41] Ho Y.S., McKay G. (1999). Pseudo-Second Order Model for Sorption Processes. Process Biochem..

[42] Weber W.J., Morris J.C. (1963). Kinetics of Adsorption on Carbon from Solutions. J. Sanit. Eng. Div. Am. Soc. Civ. Eng. (ASCE).

[43] Low M.J.D. (1960). Kinetics of Chemisorption of Gases on Solids. Chem. Rev..

[44] Al-Ghouti M.A., Da’ana D.A. (2020). Guidelines for the Use and Interpretation of Adsorption Isotherm Models: A Review. J. Hazard. Mater..

[45] Saleh T.A. (2022). Isotherm Models of Adsorption Processes on Adsorbents and Nanoadsorbents. Interface Science and Technology.

[46] Langmuir I. (1918). The adsorption of gases on plane surfaces of glass, mica and platinum. J. Am. Chem. Soc..

[47] Freundlich H. (1911). Kapillarchemie, Eine Darstellung der Chemie der Kolloide und Verwandter Gebiete. Nature.

[48] Redlich O., Peterson D.L. (1959). A Useful Adsorption Isotherm. J. Phys. Chem..

[49] De Vargas Brião G., Hashim M.A., Chu K.H. (2023). The Sips Isotherm Equation: Often Used and Sometimes Misused. Sep. Sci. Technol..

[50] Shafiq M., Alazba A.A., Amin M.T. (2023). Preparation of ZnMgAl-Layered Double Hydroxide and Rice Husk Biochar Composites for Cu(II) and Pb(II) Ions Removal from Synthetic Wastewater. Water.

[51] Ruíz-Baltazar Á.D.J., Reyes-López S.Y., Méndez-Lozano N., Medellín-Castillo N.A., Pérez R. (2024). Sustainable Zeolite–Silver Nanocomposites via Green Methods for Water Contaminant Mitigation and Modeling Approaches. Nanomaterials.

[52] Van’t Hoff J.H. (1896). Studies in Chemical Dynamics.

[53] Ezzeddine Z., Batonneau-Gener I., Pouilloux Y., Hamad H. (2016). Removal of Methylene Blue by Mesoporous CMK-3: Kinetics, Isotherms and Thermodynamics. J. Mol. Liq..

[54] El-Hamshary H., Fouda M.M.G., Moydeen M., Al-Deyab S.S. (2014). Removal of Heavy Metal Using Poly (N-Vinylimidazole)-Grafted-Carboxymethylated Starch. Int. J. Biol. Macromol..

[55] Dulman V., Lisa G., Bobu E., Asandei D., Adsorption of Certain Textile Dyes onto Chitosan (2020). Spectroscopic and Thermal Analysis. Cellul. Chem. Technol..

[56] Qin Y., Li H., Ma S., Li K., Zhang X., Hou D., Zheng X., Wang C., Lyu P., Xu S. (2022). Recovery and Utilization of Phosphorus from Fruit and Vegetable Wastewater. Sci. Rep..

[57] Agi A., Junin R., Zaidi Jaafar M., Aishah Saidina Amin N., Akhmal Sidek M., Yakasai F., Nurfaiz Mohd Faizal A., Gbadamosi A., Oseh J. (2022). Process Optimization of Reservoir Fines Trapping by Mesoporous Silica Nanoparticles Using Box-Behnken Design. Alex. Eng. J..

[58] Bu Z., Fang Y., Chen H., Zhang M., Wang F. (2025). Study on the Adsorption Properties of Organically Modified Diatomite for Methylene Blue. Sci. Rep..

[59] Debord J., Harel M., Bollinger J.-C., Chu K.H. (2022). The Elovich Isotherm Equation: Back to the Roots and New Developments. Chem. Eng. Sci..

[60] Ahmed S.S., Alwattar A.A., Ismael S.O., Zaki E., Hussein M., Casson A.J., Quayle P., Haddad A.M. (2024). Advanced Pyrene Copolymer/rGO Hydrogels for Efficient Congo Red Removal from Aqueous Systems. ChemistrySelect.

[61] Kodrić M., Reka A., Dimić Č., Tarbuk A., Đorđević D. (2020). Thermodynamic Investigation of Disperse Dyes Sorption on Polyester Fibers. Adv. Techol..

