# Supplementary material for: Design of a Novel Polyvinyl Imidazole-Based Adsorbent for Efficient Textile Dye Removal
Source: Nanomaterials (Basel). 2025 Nov 12;15(22):1708. doi: 10.3390/nano15221708 (PMC12655127; doi:10.3390/nano15221708)
Supplement: Supplementary file 1 [file nanomaterials-15-01708-s001.zip › nanomaterials-3911053-supplementary.pdf]

SUPPLEMENTARY MATERIAL

Design of a Novel Polyvinyl Imidazole-Based Adsorbent for Efficient Textile Dye Removal

Seyda Tugba Gunday[1,*], Arkan Almushikes[2], Fatmah Al Bibiy[2], Noor Alzayer[2], Lama Almedaires[2], Aljawharah Alagl[2], Ismail Anil[2], Omer Aga[2].

[1]Bioenergy Research Unit, Department of Biophysics, Institute for Research and Medical Consultations (IRMC), Imam Abdulrahman Bin Faisal University, P.O. Box 1982, Dammam 34212, Saudi Arabia.

[2]Environmental Engineering Department, College of Engineering A13, Imam Abdulrahman Bin Faisal University, P.O. Box 1982, Dammam 34212, Saudi Arabia.

[*]Correspondence: ianil@iau.edu.sa, stgunday@iau.edu.sa

List of Supplementary Tables

Table S1. Parameters and R^2 values of two-step non-linear plots of IPD model.

C_0	1st Step			2nd Step		
	k_{d1}	C_1	R^2	k_{d2}	C_2	R^2
50	5.25	-8.18	0.933	0.047	32.1	0.989
100	11.6	-12.5	0.968	0.067	63.6	0.981
500	17.9	211	0.820	0.094	311	0.989

List of Supplementary Figures

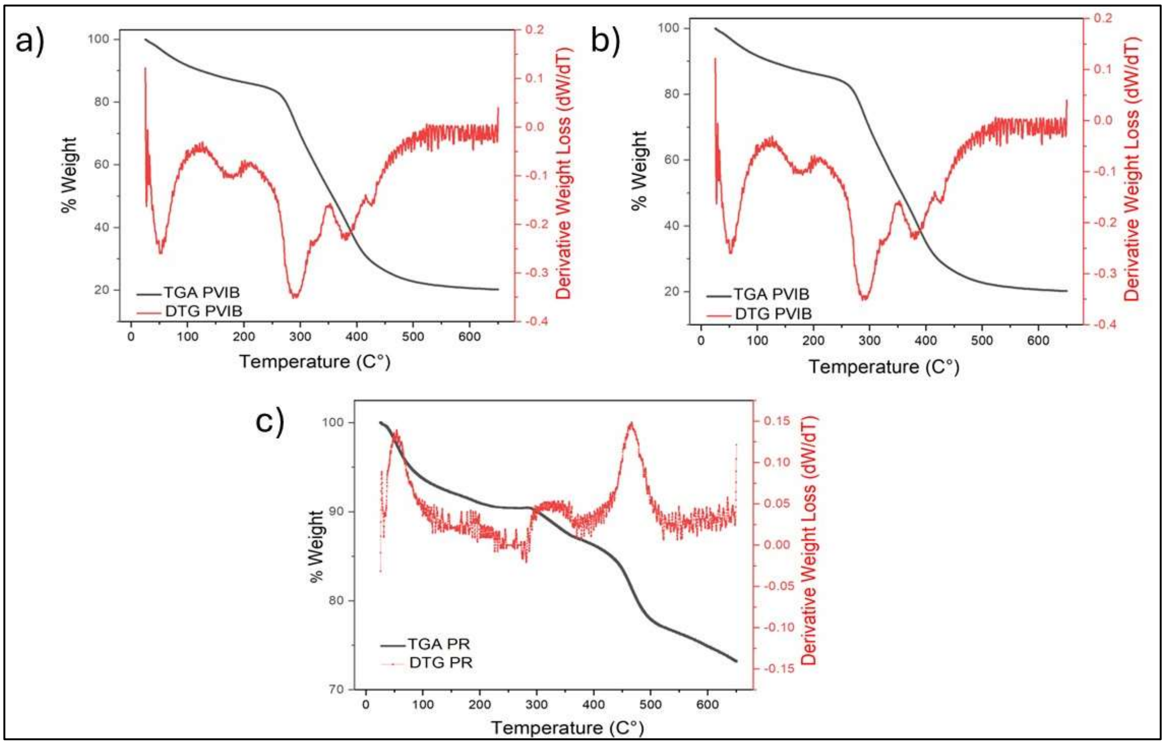

Figure S1. TGA/DTG graph of a)PVIB, b) PVIBPR, c) PR.

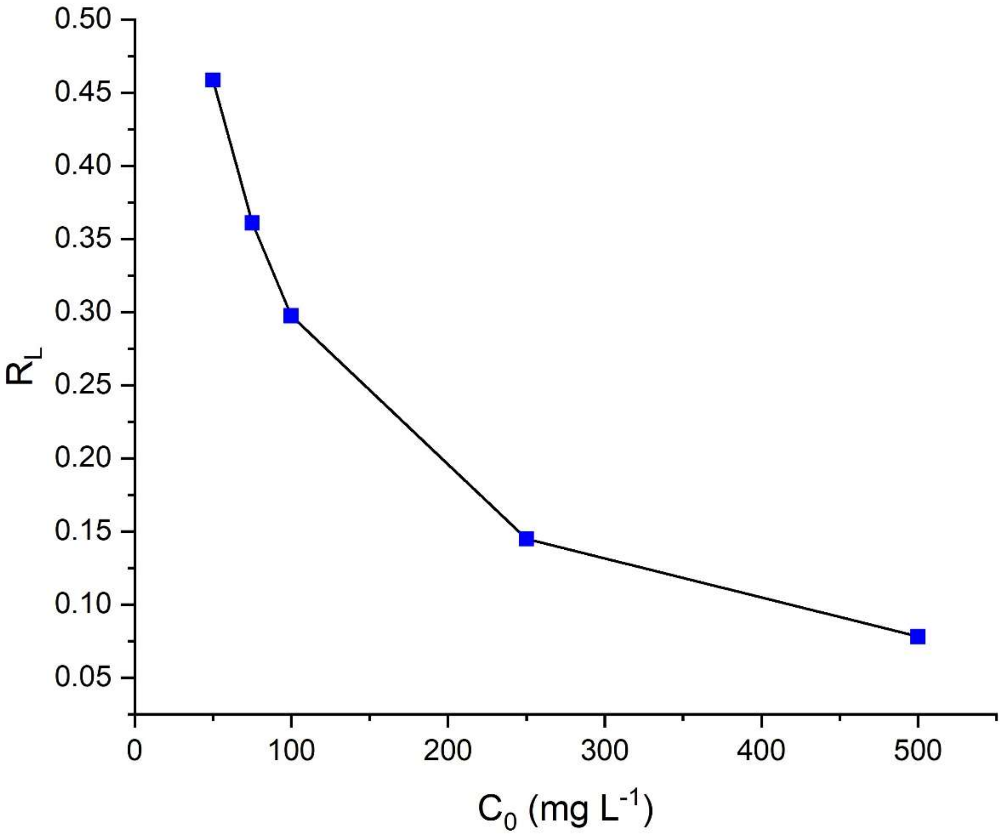

Figure S2. The change in RL value within the studied C_0 range.
